# Factors associated with influenza vaccination failure and severe disease in a French region in 2015

**DOI:** 10.1371/journal.pone.0195611

**Published:** 2018-04-17

**Authors:** Julien Marlet, Catherine Gaudy-Graffin, Daniel Marc, Ronan Boennec, Alain Goudeau

**Affiliations:** 1 French National Institute of Health and Medical Research INSERM U1259, Tours, France; 2 University of Tours, Tours, France; 3 Avian Immune Response and Pathogenesis, French National Institute for Agricultural Research INRA, Nouzilly, France; Agencia de Salut Publica de Barcelona, SPAIN

## Abstract

Current influenza vaccination strategy is based on limited analyses of circulating strains and has some drawbacks, as illustrated during the 2014–2015 season with the circulation of A(H3N2) viruses belonging to divergent genetic subgroups. We reasoned that these strains, poorly neutralized *in vitro*, may have been associated with vaccination failure and more severe diseases. We conducted a study on a continuous series of 249 confirmed influenza infections. Incidence was three fold greater than in the previous three years. Most isolates were A(H3N2) viruses (78%) and clustered in subgroups 3C.2a (57%) and 3C.3b (43%). We identified 23 non-synonymous mutations that had already been identified during previous seasons at low frequencies, except mutation Q197H, present in 26% of 3C.3b isolates. We identified lung disorder, tobacco smoking and A(H1N1)pdm09 infection as risk factor of severe influenza disease. In contrast, young age (< 5 years), A(H3N2) infection and initial admission to an emergency department were associated with a better outcome of influenza infection.

## Introduction

Influenza viruses are characterized by high genetic diversity resulting from mutations or genetic re-assortment with other influenza viruses. Influenza viruses mutate frequently, resulting in continuous antigenic drift. This permanent evolution necessitates annual updates of vaccine composition, relying on the selection of strains that antigenically match currently circulating influenza viruses (i.e. strains that induce neutralizing antibodies against circulating strains). This strategy has some drawbacks, as demonstrated during the recent 2014–2015 influenza season. The northern hemisphere influenza vaccine composition for that season was decided upon in February 2014 by the WHO (World Health Organization), based on currently circulating strains. It included the A/California/07/2009 (H1N1)pdm09, A/Texas/50/2012 (H3N2, 3C.1), and B/Massachusetts/2/2012 (Yamagata) strains. However, the circulating strains of influenza A(H3N2) evolved rapidly in 2014, with the emergence of the divergent genetic subgroups 3C.3a (reference strain A/Switzerland/9715293/2013) and 3C.2a (reference strain A/Hong Kong/5738/2014) [1]. These viruses reacted poorly in haemagglutination inhibition (HI) assays with post-infection ferret antiserum raised against the 3C.1 vaccine strain A/Texas/50/2012 and as such, were considered as antigenic drift variants [1]. At the beginning of the 2014–2015 influenza season, most circulating viruses belonged to these divergent subgroups [2]. Genetic diversity increased during this season with the emergence of a new subgroup, 3C.3b, corresponding to antigenically stable viruses (i.e. correctly neutralized in HI assays) [3].

In France, the 2014–2015 influenza season lasted nine weeks and was associated with 2.9 million consultations, 1,558 cases of severe influenza necessitating intensive care, and 18,300 excess deaths [4]. Vaccination coverage was low (53%) for patients at risk of severe influenza (i.e. patients > 65 years, patients with comorbidities (lung, heart, renal, immune or endocrine), and pregnant women). Although, they all should have been vaccinated, according to French recommendations [4]. Most people were infected with influenza A viruses (75%), corresponding to 53% A(H3N2) and 19% A(H1N1)pdm09 [5]. Limited genetic and antigenic analysis was performed on circulating viruses during that flu season by the national influenza reference center to identify variant strains. Genetic analysis revealed that variant subgroups 3C.2a and 3C.3a co-circulated in equal proportions [5]. Antigenic analysis showed that 43% of A(H3N2) isolates were antigenic drift variants, similar to the variant strain A/Switzerland/9715293/2013 (3C.3a) [5]. In the present study, we reasoned that the divergent A(H3N2) 3C.2a and 3C.3a subgroups, which poorly matched the vaccine strain *in vitro*, may have been associated with vaccination failure and more cases of severe disease. We addressed this issue by conducting a large retrospective study on a continuous series of laboratory-confirmed influenza infections. The aim was to obtain a representative overview of influenza infections in the Centre-Val de Loire region (2.6 million inhabitants, 4% of the French population). We determined the clinical profiles of patients and the genetic diversity of circulating influenza viruses and then identified the clinical and viral factors associated with severe influenza disease and factors associated with vaccination among patients with influenza A infection.

## Materials and methods

### Clinical samples

A continuous series of patients (N = 1323) were hospitalized at the Bretonneau hospital (Tours, Centre-Val de Loire region, France) between December 24, 2014 (week 52) and April 1, 2015 (week 14) with symptoms of respiratory infection (fever, cough, dyspnea), with or without headache, myalgia, or arthralgia. They had diagnostic procedures for viral respiratory tract infections. All patients with at least one respiratory sample (nasopharyngeal aspirate or bronchoalveolar lavage) positive for influenza virus were included in our study. Laboratory diagnoses were performed using multiplex Anyplex^™^ II RV16 & RB5 PCR assays (Seegene) or the Filmarray^®^ respiratory panel (Biomerieux) for emergency cases [6,7].

Patient clinical history was analyzed by reviewing the clinical chart of their medical records. Young children (< 5 years) and elderly patients (> 65 years) are known to be at high risk of complications [8] and were studied separately from the other patients [9]. Patient comorbidities were divided into pulmonary (asthma, chronic obstructive bronchopneumopathy and respiratory failure), cardiac (coronaropathy and cardiac failure), renal (renal failure), endocrine (obesity and diabetes), cancers and immune disorders (immunosuppressive treatment, HIV infection and hereditary immune deficit). Nosocomial infections were defined as those with symptom onset after 48 h of hospitalization or admission in a nursing home. The clinical presentation was considered to be atypical when initial symptoms were different from those listed above. Vaccinated patients had received the 2014–2015 influenza vaccine prior to infection. A poor outcome of influenza infection was defined as either complication, admission to an intensive care unit (ICU), or death. Complications included severe acute respiratory failure, exacerbation of chronic pneumopathy, pneumonia, sepsis, and heart, liver, or kidney failure. Antiviral treatment was defined as the prescription of oseltamivir, zanamivir, or amantadine. Data were anonymized prior to analysis in accordance with the latest version of the Helsinki Declaration of human research ethics. This study was approved by the ethic committee “Espace de Réflexion Ethique Région Centre” (approval number 2017 066).

### Molecular and phylogenetic analysis

Influenza A virus subtyping was performed by qPCR as recommended by the WHO [10]. Sanger sequencing of the hemagglutinin (HA) gene was performed to identify subgroup-specific mutations. All sequences were validated using the Influenza Research Database (IRD) annotation tool and deposited in the Genbank database (Accession numbers KY615063—KY615236), using the WHO nomenclature system [11]. Using A/Texas/50/2012 as the reference strain, mutations were analyzed with the tools available at the Influenza Research Database (https://www.fludb.org). Genetic subgroups were determined according to available CDC recommendations [3]. Potential glycosylation sites were predicted using the NetNGlyc 1.0 server (http://www.cbs.dtu.dk/services/NetNGlyc/), setting 0.5 as the threshold value of the average potential to predict glycosylation sites. Phylogenetic analyses were performed to confirm identification of genetic subgroups. Multiple alignments of HA gene nucleotide sequences were performed using the Bioedit program and MUSCLE algorithm. A neighbor-joining tree was constructed with Mega 7 software, using the Kimura 2-parameter model [12]. The influenza A(H3N2) reference strains A/Texas/50/2012 (genetic subgroup 3C.1), A/Hong Kong/146/2013 (3C.2), A/Hong Kong/5738/2014 (3C.2a), A/Samara/73/2013 (3C.3), and A/Newcastle/22/2014 (3C.3b) were included in the tree, which was rooted with A/Texas/50/2012 (vaccine strain).

### Statistical analysis

Results are expressed as the mean and standard deviation (SD) or median and interquartile range (IQR) for continuous variables, and percentage for categorical variables. Statistical analyses were performed using Epi Info 7 (CDC). The Chi squared test or Fisher’s exact test were performed for categorical variables. Student *t*-test or Mann-Whitney test were performed for continuous variables. All tests were two-sided at the 0.05 significance level. Factors associated with severe influenza infection or vaccination were identified using multivariate logistic regression adjusted for age as a potential confounding factor. Results are expressed as the odds-ratio (OR) with their 95% confidence interval (95% CI).

## Results

### Influenza virus epidemiology in a French region in 2014–2015

**Fig 1:**
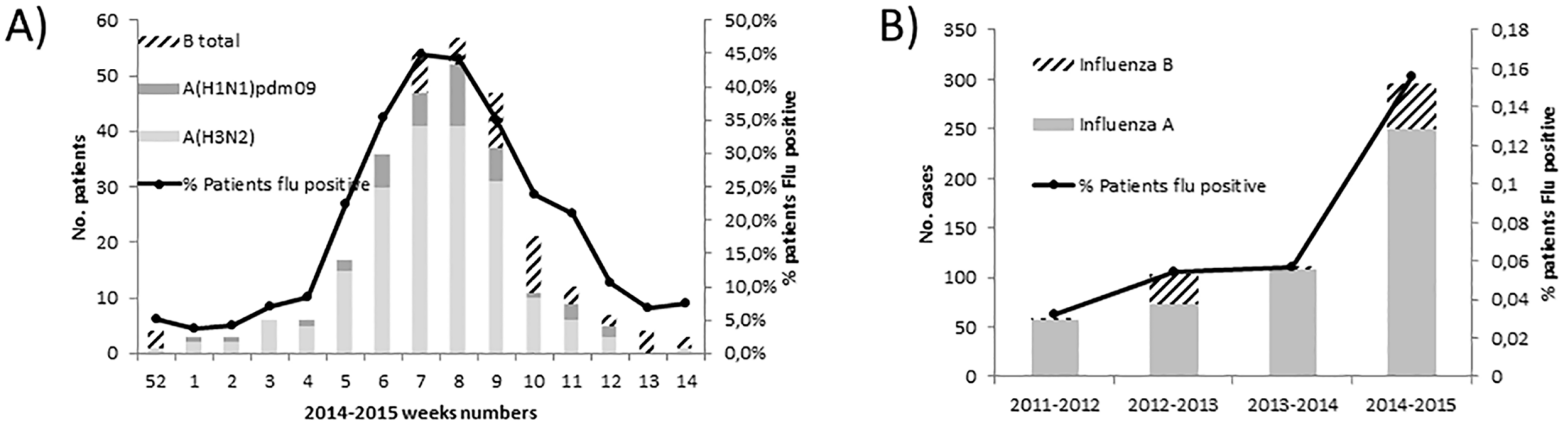
Influenza virus epidemiology in the Tours university hospital (Centre-Val de Loire region, France). (A) Weekly reports of influenza infections from week 52 of 2014 to week 14 of 2015 (N = 1323 patients). (B) Prevalence of influenza infections for the last four years between week 40 of year Y-1 and week 18 of year Y, among all patients screened for respiratory tract infections in our center.

The 2014–2015 influenza season started on week 52 of 2014 and ended on week 14 of 2015, with a peak incidence at weeks 7 and 8 (Fig 1A). Rate of influenza detection was three-fold higher than in previous years (N = 296, 16%), with similar influenza detection procedures (Fig 1B). Most of the 296 patients were infected with influenza A viruses (N = 249; 84%).

Subtyping of the 249 influenza A positive samples identified 250 influenza A viruses (because one patient was co-infected with H1N1 and H3N2 viruses). These 250 influenza A viruses were mostly A(H3N2) viruses (n = 194/249; 78%), including three viruses with H3N2-like subtyping results. The latter were H3 positive but N2 negative, probably because of poor performance of the N2-specific RT-qPCR. Other subtypes were A(H1N1)pdm09 (n = 40; 16%) and two atypical profiles (1%): one H1-/N1+ and one H3-/N2+, that we attributed to probable HA mutations at primer binding sites. Fourteen viruses were not typed, either due to an insufficient amount of biological material (n = 11; 4%) or because all subtyping RT-PCRs were negative (n = 3; 1%).

### Clinical profiles of patients with influenza virus infections

The medical records of all patients tested positive for influenza A (N = 249) were retrospectively analyzed. Demographic data, comorbidities, and details on influenza infections were extracted (Table 1). Young children were at lower risk of comorbidities (OR = 0.16, 95% CI = 0.08–0.35), admission in ICU (OR = 0.14, 95% CI = 0.04–0.51) and severe disease (OR = 0.29, 95% CI = 0.14–0.61) in comparison to patients aged 5–65 years.

**Table 1:** Clinical profiles of patients positive for influenza viruses.

	Influenza A no. (%)	A(H3N2) no. (%)	A(H1N1)pdm09 no. (%)	p value
**Total**	249 (100)	194 (100)	40 (100)	
**Age**				<0.001
< 5 years	55 (22)	38 (20)	16 (40)	0.248
5–65 years	84 (34)	61 (31)	16 (40)	-
> 65 years	110 (44)	95 (49)	8 (20)	0.009
**Sex ratio (F/M)**	0.97	0.96	1.22	0.558
**Tobacco smoking**	26 (10)	18 (9)	3 (8)	0.746
**Alcohol abuse**	14 (6)	11 (6)	2 (5)	0.888
**Comorbidity**	155 (62)	123 (63)	22 (55)	0.273
Lung disorders	53 (21)	45 (23)	6 (15)	0.164
Endocrine disorders	53 (21)	43 (22)	9 (23)	0.863
Heart disorders	40 (16)	32 (17)	5 (13)	0.466
Cancers & Immune disorders	45 (18)	35 (18)	6 (15)	0.681
**Department of admission**				0.005
Emergency	30 (12)	23 (12)	6 (15)	0.204
ICU	47 (19)	29 (15)	14 (35)	0.002
Other	172 (69)	142 (73)	20 (50)	-
**Days of hospitalization**^a^	5 (2–10)	5 (1.5–9)	4.5 (2–12)	0.604
**Nosocomial influenza**	9 (4)	7 (4)	0	0.605
**Atypical presentation**	36 (15)	31 (16)	4 (10)	0.466
**Co-infection**	52 (21)	39 (20)	12 (30)	0.146
**Poor outcome**	141 (57)	106 (55)	25 (63)	0.413
Complication	138 (55)	103 (53)	25 (63)	0.321
ICU hospitalization	59 (24)	36 (19)	16 (40)	0.002
Death	16 (6)	11 (6)	3 (8)	0.710
**Influenza vaccine**	26 (10)	23 (12)	1 (2.5)	0.090
**Antiviral treatment**	39 (16)	26 (13)	8 (20)	0.257

^a^Results expressed as median and interquartile range (IQR) for continuous variables

In contrast, elderly patients were at higher risk of comorbidities (OR = 2.00, 95% CI = 1.05–3.79) but at lower risk of atypical infections (OR = 0.23, 95% CI = 0.09–0.61) and they were more frequently vaccinated (OR = 3.30, 95% CI = 1.18–9.24) in comparison to patients aged 5–65 years. Also, elderly patients were at higher risk of infection with A(H3N2) (OR = 2.39, 95% CI = 1.16–4.94) and at lower risk of infection with A(H1N1)pdm09 (OR = 0.33, 95% CI = 0.14–0.82) in comparison to patients aged 5–65 years.

The clinical presentation was atypical for 36 patients (14%), which had no symptoms of respiratory infection, but presented with asthma (n = 5), asthenia (n = 4) or seizures (n = 4).

### Genetic diversity of influenza A(H3N2) viruses

**Fig 2:**
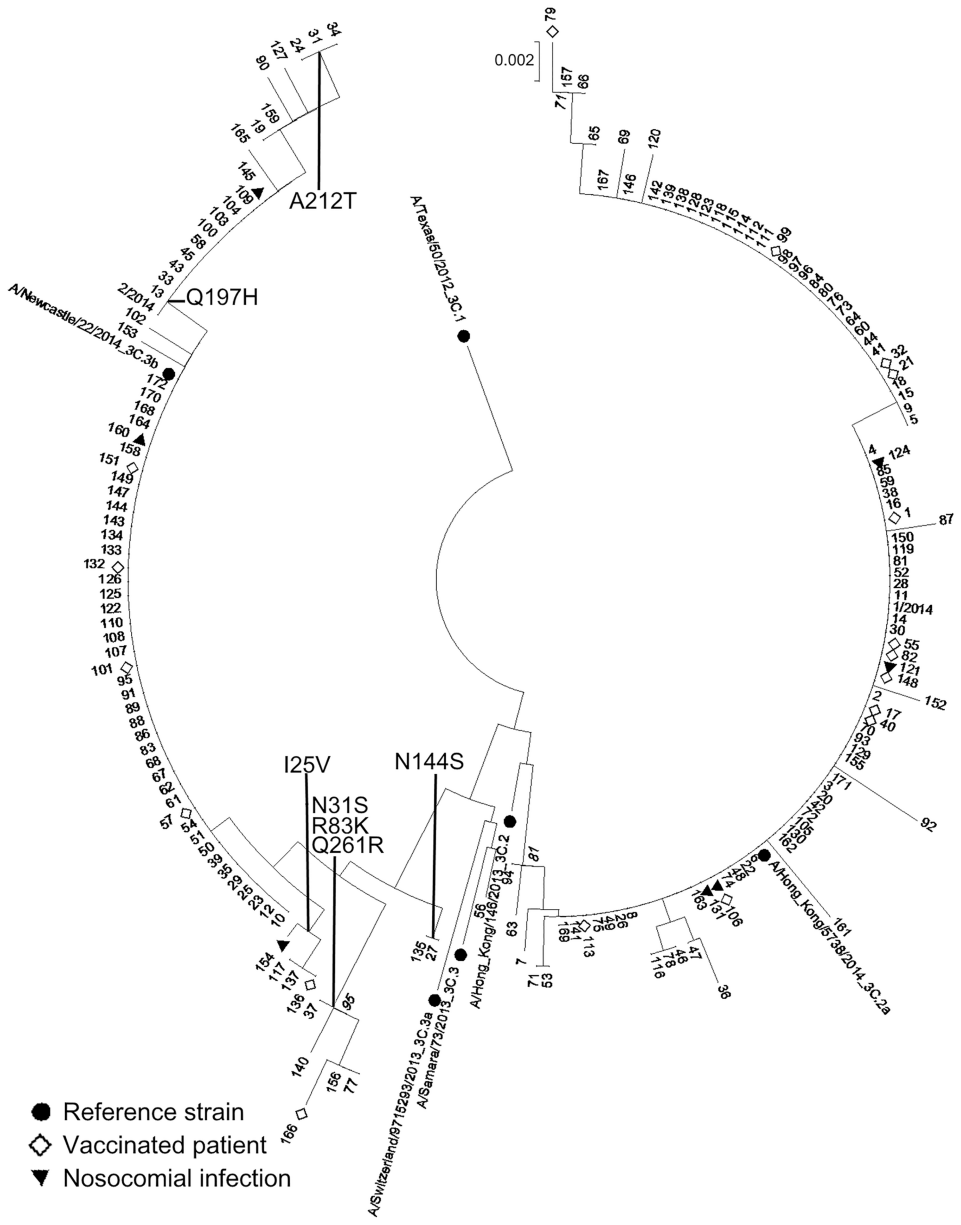
Rooted phylogenetic tree of the HA gene for the 2014–2015 influenza A(H3N2) viruses. The evolutionary history was inferred using the neighbor-joining method [13]. The optimal tree with the sum of branch length = 0.13659130 is shown. The tree is drawn to scale, with branch lengths in the same units as those of the evolutionary distances used to infer the phylogenetic tree. The evolutionary distances were computed using the Kimura 2-parameter method [14] and are expressed as the number of base substitutions per site. The analysis involved 180 nucleotide sequences. All positions containing gaps and missing data were eliminated. There were a total of 395 positions in the final dataset. Evolutionary analyses were conducted using MEGA7 [15]. Bootstrap values greater than 70% are displayed on branch nodes (inferred from 1000 replicates). Amino-acid substitutions delineating major branches are shown. Sequences A/Tours/Strain number/2015 (H3N2) are identified only by their strain number on the tree. HA sequence from reference strain A/Switzerland/9715293/2013 used in the genetic analysis was obtained from the EpiFlu database of the Global Initiative on Sharing Avian Influenza Data (GISAID, www.gisaid.org). The authors gratefully acknowledge the originating and submitting laboratories.

We sequenced the HA gene of 174 (90%) out of 194 A(H3N2) isolates to construct a phylogenetic tree (Fig 2). We observed an outbreak of influenza infection in a nursing home for the elderly, suggesting nosocomial transmission. The viruses from four of these patients shared an identical HA sequence (isolates 106, 121, 124 and 131 in Fig 2), which support the hypothesis of nosocomial transmission. Among these four patients, one was vaccinated and one died of flu-related complications. We observed a genetic drift between the A/Texas/50/2012 vaccine strain (3C.1) and the 2014–2015 isolates, with a mean genetic distance of 2.09% (1.60–2.80%). Such a genetic drift contributed to the diversity of the A(H3N2) strains which clustered in two genetic subgroups, 3C.2a (n = 99; 57%) and 3C.3b (n = 74; 43%), whereas only one 3C.3 strain was identified (isolate 56 in Fig 2). Analysis of the HA mutations revealed that five of the 74 3C.3b isolates lacked the K83R subgroup-specific mutation, with no predicted impact on glycosylation. In addition to the well-characterized subgroup-specific mutations, we identified 23 other non-synonymous mutations (Table 2) that were already identified during previous seasons. All 23 mutations were detected at low frequencies, except mutation Q197H, which was found in 19/74 (26%) of the 3C.3b isolates (Fig 2). This mutation was not associated with poor outcome and had no predicted impact on a potential glycosylation site.

**Table 2:** Additional HA mutations in the three genetic subgroups.

Additional HA mutations (no. samples)		
3C.2a strains (99)	3C.3b strains (74)	3C.3 strains (1)
None (83)	None (42)	None (0)
I29V (1)	G5E (4)	S124N^a^ (1)
D53N (1)	T12M (1)	-
D53G (1)	I25V (5)	-
S54I (2)	N31S (4)	-
V166M (1)	S54I (2)	-
E172G (1)	F79L (1)	-
Q197K (1)	Q197H (19)	-
R208G (1)	A212T (4)	-
S209N (1)	S219F (1)	-
I214T (2)	S262N (3)	-
R222K (1)	-	-
I230V (1)	-	-
R261L (1)	-	-
S262N (3)		-

^a^Loss of a potential glycosylation site

### Clinical and viral factors associated with severe influenza disease

A subset of 141 (57%) patients had a poor outcome of influenza infection (Table 1). Among them, 101 (72%) had at least one comorbidity and 75 (54%) were elderly. Univariate analysis identified young age (< 5 years) as a variable associated with better outcome of influenza infection (OR = 0.29, 95% CI = 0.14–0.62) (Table 3). Consequently, we performed an age-stratified multivariate analysis, which allowed us to identify several factors associated with a poor outcome of influenza virus infection: lung disorders (OR = 2.55, 95% CI = 1.26–5.15), tobacco smoking (OR = 4.88, 95% CI = 1.54–15.47), H1N1 infection (OR = 2.26, 95% CI = 1.03–4.95) and antiviral treatment (OR = 2.59, 95% CI = 1.11–6.05) (Table 3). In contrast, two factors were associated with better outcome: H3N2 infection (OR = 0.49, 95% CI = 0.25–0.97) and initial admission to an emergency department (OR = 0.10, 95% CI = 0.02–0.46). There was no significant association between severe infection and vaccination status, genetic A(H3N2) subgroup, sex, alcohol abuse, heart disorders, immune disorders or endocrine disorders, or atypical infections and co-infections.

**Table 3:** Factors associated with severe disease and vaccination.

	Poor outcome	Influenza vaccination
	Adjusted OR^a^ (95% CI^b^^)^	Adjusted OR^a^ (95% CI^b^^)^
**Age**		
< 5 years	**0.29 (0.14–0.61)**	0.60 (0.11–3.18)
5–65 years	1.00	1.00
> 65 years	1.60 (0.88–2.89)	**3.30 (1.18–9.24)**
**Sex ratio (F/M)**	0.86 (0.51–1.46)	**0.38 (0.16–0.92)**
**Tobacco smoking**	**4.88 (1.54–15.47)**	0.93 (0.19–4.59)
**Alcohol abuse**	1.59 (0.47–5.35)	2.88 (0.69–12.04)
**Comorbidity**	1.74 (0.97–3.14)	**14.75 (1.83–118.75)**
Lung disorders	**2.55 (1.26–5.15)**	2.30 (0.93–5.70)
Endocrine disorders	1.04 (0.53–2.03)	1.23 (0.48–3.11)
Heart disorders	1.10 (0.51–2.35)	1.72 (0.66–4.48)
Cancers & Immune disorders	0.69 (0.35–1.36)	0.71 (0.23–2.21)
Kidney disorders	**0.35 (0.15–0.82)**	0.76 (0.21–2.82)
**Department of admission**		
Emergency	**0.10 (0.02–0.46)**	0.00 (0.00–1.0.10^12^)
ICU	-	1.25 (0.46–3.42)
Other	1.00	1.00
**Nosocomial influenza**	0.35 (0.09–1.37)	0.64 (0.08–5.41)
**Atypical presentation**	1.02 (0.48–2.20)	1.27 (0.33–4.80)
**Co-infection**	1.87 (0.94–3.74)	0.88 (0.28–2.78)
**Hospitalization > 5 days**	**6.33 (3.45–11.62)**	0.94 (0.40–2.23)
**Poor outcome**	-	1.08 (0.44–2.64)
Complication	-	1.12 (0.46–2.73)
ICU hospitalization	-	1.62 (0.64–4.12)
Death	-	0.83 (0.17–3.97)
**Influenza vaccine**	1.08 (0.44–2.64)	-
**Antiviral treatment**	**2.59 (1.11–6.05)**	1.10 (0.38–3.20)
**H3N2 (vs H1N1)**	**0.44 (0.20–0.98)**	3.28 (0.41–26.00)
**Subgroup 3C.3b (vs 3C.2a)**	1.39 (0.73–2.64)	1.12 (0.42–3.02)
**Q197H HA mutation**	0.94 (0.31–2.87)	0.35 (0.04–3.08)

^a^Adjusted OR, odds-ratio adjusted for age. Values in bold, p-value<0.05

^b^CI, confidence interval

### Factors associated with vaccination among patients with influenza A infection

Among the 249 patient with influenza A infection, 10% (n = 26) had been vaccinated earlier in the current season. Most of these patients were > 65 years old (n = 19; 73%), male (n = 18; 69%), had at least one comorbidity (n = 25; 96%) and were initially admitted to a clinical department other than emergency or ICU (n = 20; 77%). Twenty-three (88%) were infected with an A(H3N2) strain (11 with 3C.2a strains and 8 with 3.C3b strains) and one with an A(H1N1)pdm09 strain (two samples were missing).

Univariate analysis performed on these 249 influenza cases identified a higher prevalence of elderly patients (> 65 years) in the vaccinated group than in the non-vaccinated group (OR = 3.30, 95% CI = 1.18–9.24) (Table 3). Therefore, we performed an age-stratified multivariate analysis and identified several other factors associated with the vaccinated patients (Table 3): comorbidities (OR = 14.75, 95% CI = 1.83–118.75) and female patients (OR = 0.38, 95% CI = 0.16–0.92). Apart from these two clinical factors, no other clinical factors (co-infection, department of admission, tobacco smoking, alcohol abuse, or severe infection), neither viral factors (viral subtype, genetic subgroup nor emerging mutations like Q197H) were associated with vaccinated patients.

## Discussion

We accurately described both clinical and virological features of the 2014–2015 influenza epidemic, at a regional scale, on a continuous series of 249 hospitalized patients with influenza A infection. In comparison with previous years, rate of influenza detection was three-fold higher, confirming the increased incidence this year [16]. Most patients (78%) were infected with A(H3N2) viruses. A(H3N2) viruses were characterized by a genetic drift from the A/Texas/50/2012 vaccine strain (3C.1). These viruses clustered in antigenically divergent subgroup 3C.2a and antigenically stable subgroup 3C.3b. A poor outcome was more frequent for patients with lung disorder, those smoking tobacco, or those infected with A(H1N1)pdm09. In contrast, young age (< 5 years), A(H3N2) infection and initial admission to an emergency department were associated with a better outcome of influenza infection.

When A(H1N1)pdm09 emerged in 2009, mortality caused by this strain was similar to that of seasonal influenza with a mean hospitalization fatality rate (HFR) of 3%, but elderly patients (> 65 years) were at a lower risk of infection than adults and children [17–20]. We confirmed these findings for A(H1N1)pdm09 with a HFR of 7.5%, and a lower rate of infection for elderly patients (OR = 0.33, 95% CI = 0.14–0.82) in comparison to patients aged 5–65 years. For these patients born before 1950, first influenza virus infection probably was H1 or H2 (circulating viruses until 1968), both belonging to HA phylogenetic group 1 [21]. Recent work suggests that this first infection drives an HA antigenic imprinting which confers lifelong protection against viruses of the same phylogenetic group and could explain our results [21].

The 174 HA sequences obtained in this study and their associated clinical features contribute to a better understanding of the genetic diversity A(H3N2) virus in France and the northern hemisphere. Our regional results are in agreement with European data, with 3C.2a and 3C.3b being the most prevalent subgroups [3,5]. These 174 HA sequences will add to the 93 sequences published so far for the 2014–2015 season in Europe (32 from one French region and 54 from Czech Republic).

The 2014–2015 influenza season in France started with a high risk of vaccination failure because of the predominant circulation of variant strains [2]. This risk was further increased by the low vaccination coverage (53%) of patients at risk of severe influenza disease. Vaccination failure was confirmed by the low estimated vaccine effectiveness (VE) in France, especially for elderly patients > 65 years (VE = 5%, 95% CI = -8-16%) [22]. Our study tends to confirm this poor VE, with a prevalence of vaccinated patients among influenza cases in our region of 10%, similar to the reported 8.7% in France [5].

Among patients with influenza disease, this study identified that elderly patients [23] (OR = 3.30, 95% CI = 1.18–9.24), comorbidities (OR = 14.75, 95% CI = 1.83–118.75) and female patients (OR = 0.38, 95% CI = 0.16–0.92) were associated with influenza vaccination. These results are compatible with previous studies which identified age as risk factor of vaccination failure [23] and female patients as better vaccine responders than male patients [24]. Of note, the association between vaccination and elderly patients or patients with comorbidities may be due to their over-representation in the vaccinated group, because they are targeted by vaccination campaigns. Further studies including more patients and a control group with influenza negative patients are needed to determine if these factors are associated with vaccination failure.

The multisite US Flu Vaccine Effectiveness Network estimated the 2014–2015 influenza vaccine effectiveness (VE) to be 23% (95% CI = 8–36%). This is far below the 50% estimated for the previous seasons. This suggests that, apart from clinical factors, several viral factors could be responsible for this low VE [25,26].

The most important viral factor is probably the predominant circulation of 3C.2a viruses [3,25], poorly neutralized in HI assay [2]. A recent study demonstrated that VE in the USA was significantly lower for the 3C.2a subgroup (VE = 1%, 95% CI = -14-14%) than for the 3C.3b subgroup (VE = 44%, 95% CI = 16–63%) [25]. A possible explanation for that is that recent H3N2 viruses in the 3C.2a subgroup possess a new glycosylation site in antigenic site B of HA (mutation K160T), associated with poor neutralization by vaccine induced antibodies [27]. These 3C.2a viruses could have been associated with influenza vaccination failure in France [22], as they represented 57% of all A(H3N2) isolates, and they all possessed the K160T mutation.

Strains belonging to 3C.3a subgroup, poorly neutralized in HI assay [2], could also be responsible for vaccination failure but they were not observed in this study and thus cannot account for vaccination failure in France, in the Centre-Val de Loire region.

A third potential factor to explain vaccination failure is HA mutation Q197H/R, located in epitope B and identified as a critical position for recognition by neutralizing-antibodies [28]. Mutation Q197H has been observed since 2015 only in 3C.3b viruses in Germany [29]. Mutation Q197R was observed in 2015 only in 3C.2a viruses in the USA [25]. In this French study, mutation Q917H was found in 26% (19/74) of the 3C.3b viruses and we described for the first time a new but similar mutation (electrically charged side chain amino acid), Q197K in one 3C.2a virus. We hypothesize that these HA mutations at residue 197 could be associated with vaccination failures and need to be monitored in future influenza seasons.

In addition to these viral factors, impaired immune response to influenza vaccine probably contributed to vaccination failure. Screening methods for evaluating new influenza vaccines relies mostly on antibody responses [30]. Yet, antibody responses are considerably lower in the elderly than in younger adults [23,31]. T-cells responses have been reported to better correlate with protection in the elderly [30]. Thus, poor T-cells responses to seasonal influenza vaccines could have contributed to vaccination failure, especially in the elderly.

Overall, the circulation of antigenic drift variant 3C.2a strains remains the most likely explanation for vaccination failure in the northern hemisphere during the 2014–2015 influenza season [2,25].

With three to five million cases of severe influenza-related illness every year and low VE (23–50%), there is an evident need to focus our efforts on more effective surveillance and prevention strategies [26]. The current official strategy in France is focused on patients at risk of severe influenza disease, but this goal was not reached, with a vaccination coverage of only 53% for these patients and a VE of only 5% (95% CI = -8-16) for patients > 65 years [22]. In addition, the mean influenza VE since 2004 has remained too low (41%, 10–60%) [26]. Such a low VE would not be acceptable for many vaccines and carries a cost [32]. There is a pressing need for an increased global investment on influenza vaccine research [33]. Finally, an acceptable VE requires an accurate and rapid selection of strains able to induce neutralizing antibodies against currently circulating strains. To do so, WHO and National Influenza Centers (NICs) must receive enough samples in time to provide an exhaustive overview of influenza virus evolution. In this perspective, we described an emerging HA mutation at a critical position, Q197H, which need to be monitored because its association with other mutations could impair vaccine effectiveness. Also, we highlighted the importance of 3C.3b subgroup (43% of circulating strains) for vaccination strategies in our region, while the French 2015 national influenza report did not mention this subgroup. This illustrates that influenza virus evolution may vary greatly in a given country [25]. Such viral evolution analysis is important to detect emergent and divergent influenza strains at their very beginning. This early detection is crucial to adapt next season influenza vaccine in time to prevent vaccination failure associated with emergence of divergent strains. For this reason, it is crucial that NICs rely on a tight network of local collaborating laboratories.

## References

[1] ECDC, ERLI-Net. Influenza Virus Characterisation. Summary Europe, September 2014. 2014 Sep.

[2] ECDC, ERLI-Net. Influenza Virus Characterisation. Summary Europe, November 2014 [Internet]. 2014 Nov. http://ecdc.europa.eu/en/publications/Publications/ERLI-Net%20report%20November%202014.pdf

[3] ECDC, ERLI-Net. Influenza Virus Characterisation. Summary Europe, April 2015 [Internet]. 2015 Apr. http://ecdc.europa.eu/en/publications/Publications/influenza-virus-characterisation-april-2015.pdf

[4] INVS. Bilan de la saison grippale 2014–2015 [in French]. 2015 May.

[5] Van der Werf S. Surveillance virologique de la grippe: saison 2014–2015. [in French]. Bull Epidémiol Hebd. 10 2015: 599–603.

[6] Poritz MA, Blaschke AJ, Byington CL, Meyers L, Nilsson K, Jones DE, et al FilmArray, an Automated Nested Multiplex PCR System for Multi-Pathogen Detection: Development and Application to Respiratory Tract Infection. Costa C, editor. PLoS ONE. 2011;6: e26047 doi: 10.1371/journal.pone.0026047 22039434 10.1371/journal.pone.0026047 PMC3198457

[7] Kim H-K, Oh S-H, Yun KA, Sung H, Kim M-N. Comparison of Anyplex II RV16 with the xTAG Respiratory Viral Panel and Seeplex RV15 for Detection of Respiratory Viruses. J Clin Microbiol. 2013;51: 1137–1141. doi: 10.1128/JCM.02958-12 23363824 10.1128/JCM.02958-12 PMC3666760

[8] Bonmarin I, Belchior E, Bergounioux J, Brun-Buisson C, Mégarbane B, Chappert JL, et al Intensive care unit surveillance of influenza infection in France: the 2009/10 pandemic and the three subsequent seasons. Eurosurveillance. 2015;20 doi: 10.2807/1560-7917.ES.2015.20.46.30066 26607262 10.2807/1560-7917.ES.2015.20.46.30066

[9] People at High Risk of Developing Flu–Related Complications | Seasonal Influenza (Flu) | CDC [Internet]. [cited 21 Jan 2017]. https://www.cdc.gov/flu/about/disease/high_risk.htm

[10] WHO. WHO information for molecular diagnosis of influenza virus in humans—update [Internet]. 2015. http://www.who.int/influenza/resources/documents/molecular_diagnosis_influenza_virus_humans_update_201108.pdf

[11] Memorandum W. A revision of the system of nomenclature for influenza viruses: a WHO memorandum. Bull World Health Organ. 1980;58: 585–91. 6969132 PMC2395936

[12] Tamura K, Stecher G, Peterson D, Filipski A, Kumar S. MEGA6: Molecular Evolutionary Genetics Analysis version 6.0. Mol Biol Evol. 2013;30: 2725–2729. doi: 10.1093/molbev/mst197 24132122 10.1093/molbev/mst197 PMC3840312

[13] Saitou N, Nei M. The neighbor-joining method: a new method for reconstructing phylogenetic trees. Mol Biol Evol. 1987;4: 406–425. doi: 10.1093/oxfordjournals.molbev.a040454 3447015 10.1093/oxfordjournals.molbev.a040454

[14] Kimura M. A simple method for estimating evolutionary rates of base substitutions through comparative studies of nucleotide sequences. J Mol Evol. 1980;16: 111–120. 7463489 10.1007/BF01731581

[15] Kumar S, Stecher G, Tamura K. MEGA7: Molecular Evolutionary Genetics Analysis Version 7.0 for Bigger Datasets. Mol Biol Evol. 2016;33: 1870–1874. doi: 10.1093/molbev/msw054 27004904 10.1093/molbev/msw054 PMC8210823

[16] Bonmarin I. Surveillance de la grippe en France métropolitaine. Saison 2014–2015 [in French]. 10 2015: 593–598.

[17] Reed C, Chaves SS, Perez A, D’Mello T, Daily Kirley P, Aragon D, et al Complications Among Adults Hospitalized With Influenza: A Comparison of Seasonal Influenza and the 2009 H1N1 Pandemic. Clin Infect Dis. 2014;59: 166–174. doi: 10.1093/cid/ciu285 24785230 10.1093/cid/ciu285 PMC7314251

[18] Lehners N, Geis S, Eisenbach C, Neben K, Schnitzler P. Changes in Severity of Influenza A(H1N1)pdm09 Infection from Pandemic to First Postpandemic Season, Germany. Emerg Infect Dis. 2013;19 doi: 10.3201/eid1905.130034 23697801 10.3201/eid1905.130034 PMC3647517

[19] Lemaitre M, Carrat F. Comparative age distribution of influenza morbidity and mortality during seasonal influenza epidemics and the 2009 H1N1 pandemic. BMC Infect Dis. 2010;10: 162 doi: 10.1186/1471-2334-10-162 20534113 10.1186/1471-2334-10-162 PMC2896934

[20] Wong JY, Kelly H, Cheung C-MM, Shiu EY, Wu P, Ni MY, et al Hospitalization Fatality Risk of Influenza A(H1N1)pdm09: A Systematic Review and Meta-Analysis. Am J Epidemiol. 2015;182: 294–301. doi: 10.1093/aje/kwv054 26188191 10.1093/aje/kwv054 PMC4528954

[21] Gostic KM, Ambrose M, Worobey M, Lloyd-Smith JO. Potent protection against H5N1 and H7N9 influenza via childhood hemagglutinin imprinting. Science. 2016;354: 722–726. doi: 10.1126/science.aag1322 27846599 10.1126/science.aag1322 PMC5134739

[22] INVS, UPMC, INSERM. Bulletin du réseau Sentinelles du 15/04/15, n° 2015s15 [in French] [Internet]. 2015 Apr. http://websenti.u707.jussieu.fr/sentiweb/3067.pdf

[23] Song JY, Cheong HJ, Hwang IS, Choi WS, Jo YM, Park DW, et al Long-term immunogenicity of influenza vaccine among the elderly: Risk factors for poor immune response and persistence. Vaccine. 2010;28: 3929–3935. doi: 10.1016/j.vaccine.2010.03.067 20394719 10.1016/j.vaccine.2010.03.067

[24] Klein SL, Pekosz A. Sex-based Biology and the Rational Design of Influenza Vaccination Strategies. J Infect Dis. 2014;209: S114–S119. doi: 10.1093/infdis/jiu066 24966191 10.1093/infdis/jiu066 PMC4157517

[25] Flannery B, Zimmerman RK, Gubareva LV, Garten RJ, Chung JR, Nowalk MP, et al Enhanced Genetic Characterization of Influenza A(H3N2) Viruses and Vaccine Effectiveness by Genetic Group, 2014–2015. J Infect Dis. 2016;214: 1010–1019. doi: 10.1093/infdis/jiw181 27190176 10.1093/infdis/jiw181 PMC5812259

[26] CDC. Seasonal Influenza Vaccine Effectiveness, 2005–2016 [Internet]. https://www.cdc.gov/flu/professionals/vaccination/effectiveness-studies.htm

[27] Zost SJ, Parkhouse K, Gumina ME, Kim K, Diaz Perez S, Wilson PC, et al Contemporary H3N2 influenza viruses have a glycosylation site that alters binding of antibodies elicited by egg-adapted vaccine strains. Proc Natl Acad Sci U S A. 2017;114: 12578–12583. doi: 10.1073/pnas.1712377114 29109276 10.1073/pnas.1712377114 PMC5703309

[28] Huang J-W, Yang J-M. Changed epitopes drive the antigenic drift for influenza A (H3N2) viruses. BMC Bioinformatics. 2011;12: S31 doi: 10.1186/1471-2105-12-S1-S31 21342562 10.1186/1471-2105-12-S1-S31 PMC3044287

[29] Mostafa A, Abdelwhab E-SM, Slanina H, Hussein MA, Kuznetsova I, Schüttler CG, et al Phylogenetic analysis of human influenza A/H3N2 viruses isolated in 2015 in Germany indicates significant genetic divergence from vaccine strains. Arch Virol. 2016;161: 1505–1515. doi: 10.1007/s00705-016-2815-x 26973232 10.1007/s00705-016-2815-x

[30] McElhaney JE, Xie D, Hager WD, Barry MB, Wang Y, Kleppinger A, et al T cell responses are better correlates of vaccine protection in the elderly. J Immunol Baltim Md 1950. 2006;176: 6333–6339. 10.4049/jimmunol.176.10.6333 16670345

[31] Goodwin K, Viboud C, Simonsen L. Antibody response to influenza vaccination in the elderly: a quantitative review. Vaccine. 2006;24: 1159–1169. doi: 10.1016/j.vaccine.2005.08.105 16213065 10.1016/j.vaccine.2005.08.105

[32] Molinari N-AM, Ortega-Sanchez IR, Messonnier ML, Thompson WW, Wortley PM, Weintraub E, et al The annual impact of seasonal influenza in the US: Measuring disease burden and costs. Vaccine. 2007;25: 5086–5096. doi: 10.1016/j.vaccine.2007.03.046 17544181 10.1016/j.vaccine.2007.03.046

[33] Wong S-S, Webby RJ. Traditional and New Influenza Vaccines. Clin Microbiol Rev. 2013;26: 476–492. doi: 10.1128/CMR.00097-12 23824369 10.1128/CMR.00097-12 PMC3719499

